# Supplementary material for: GRIN2B disease-associated mutations disrupt the function of BK channels and NMDA receptor signaling nanodomains
Source: J Gen Physiol. 2025 Aug 5;157(5):e202513799. doi: 10.1085/jgp.202513799 (PMC12324158; doi:10.1085/jgp.202513799)

Source images for western blots shown in Fig.5A

GluN2B

Left panel

Right panel

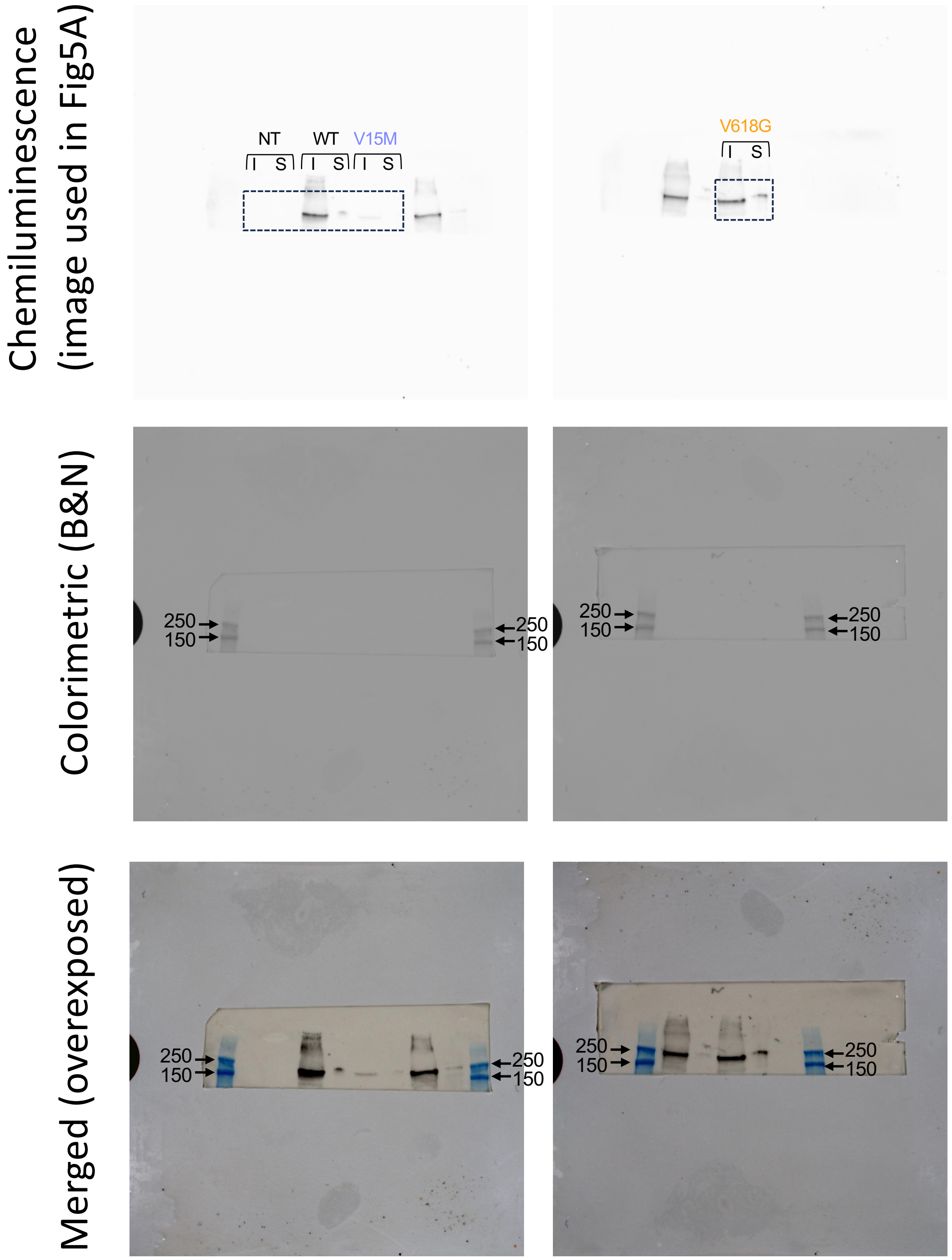

Source images for western blots shown in Fig.5A

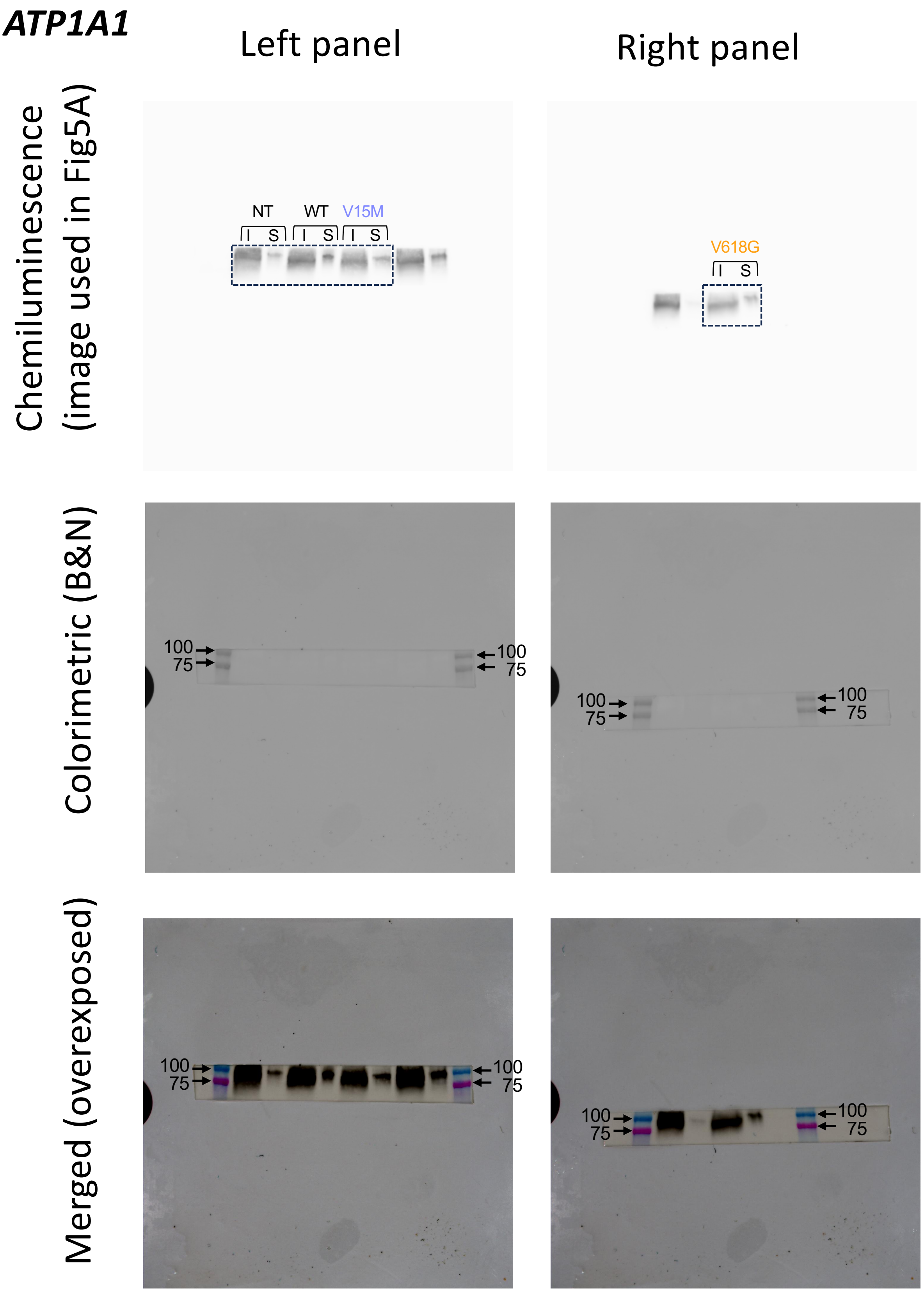

Source images for western blots shown in Fig.5A

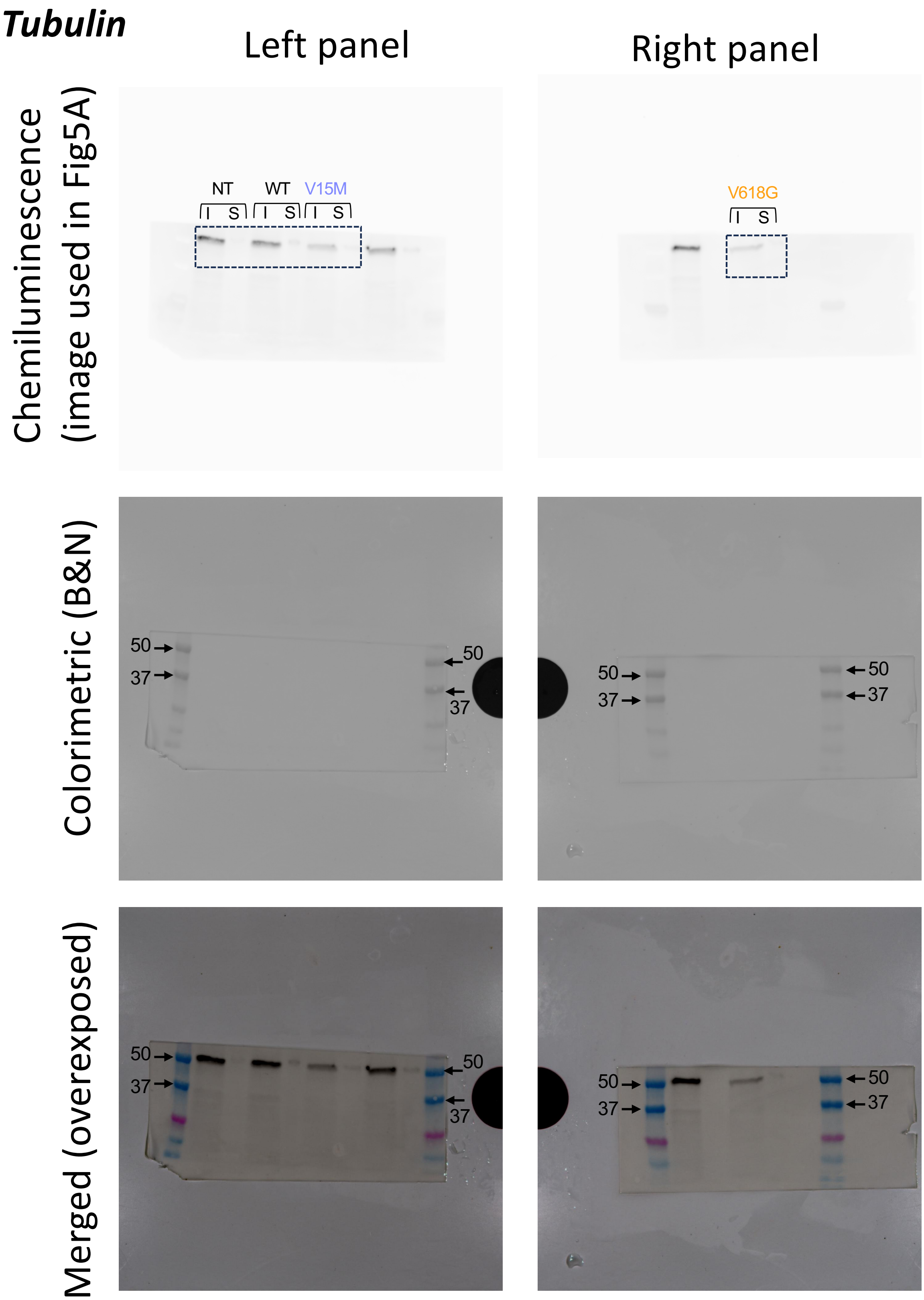

Supplement: SourceData F5 — is the source file for Fig. 5. [file jgp_202513799_sourcedataf5.pdf]
